# Development and Validation of a Targeted ‘Liquid’ NGS Panel for Treatment Customization in Patients with Metastatic Colorectal Cancer

**DOI:** 10.3390/diagnostics11122375

**Published:** 2021-12-16

**Authors:** Myrto Kastrisiou, George Zarkavelis, Anastasia Kougioumtzi, Prodromos Sakaloglou, Charilaos Kostoulas, Ioannis Georgiou, Anna Batistatou, George Pentheroudakis, Angeliki Magklara

**Affiliations:** 1 Department of Clinical Chemistry, Faculty of Medicine, University of Ioannina, 45110 Ioannina, Greece; myrto.kastrisiou@gmail.com (M.K.); natkoug@gmail.com (A.K.); 2 Department of Medical Oncology, University General Hospital of Ioannina, 45500 Ioannina, Greece; gzarkavelis@outlook.com; 3 Society for Study of Clonal Heterogeneity of Neoplasia (EMEKEN), 45444 Ioannina, Greece; 4 Laboratory of Medical Genetics in Clinical Practice, Faculty of Medicine, University of Ioannina, 45110 Ioannina, Greece; pr.sakaloglou@gmail.com (P.S.); chkostoulas@gmail.com (C.K.); igeorgio@uoi.gr (I.G.); 5 Department of Pathology, Faculty of Medicine, University of Ioannina, 45500 Ioannina, Greece; abatista@uoi.gr; 6 Institute of Molecular Biology and Biotechnology, Foundation for Research and Technology, 45110 Ioannina, Greece; 7 Institute of Biosciences, University Research Center of Ioannina (URCI), 45110 Ioannina, Greece

**Keywords:** liquid biopsies, circulating tumor DNA (ctDNA), next-generation sequencing (NGS), metastatic colorectal cancer, targeted sequencing, digital polymerase chain reaction (digital PCR)

## Abstract

The detection of actionable mutations in tumor tissue is a prerequisite for treatment customization in patients with metastatic colorectal cancer (mCRC). Analysis of circulating tumor DNA (ctDNA) for the identification of such mutations in patients’ plasma is an attractive alternative to invasive tissue biopsies. Despite having the high analytical sensitivity required for ctDNA analysis, digital polymerase chain reaction (dPCR) technologies can only detect a very limited number of hotspot mutations, whilst a broader mutation panel is currently needed for clinical decision making. Recent advances in next-generation sequencing (NGS) have led to high-sensitivity platforms that allow screening of multiple genes at a single assay. Our goal was to develop a small, cost- and time-effective NGS gene panel that could be easily integrated in the day-to-day clinical routine in the management of patients with mCRC. We designed a targeted panel comprising hotspots in six clinically relevant genes (*KRAS*, *NRAS*, *MET*, *BRAF*, *ERBB2* and *EGFR*) and validated it in a total of 68 samples from 30 patients at diagnosis, first and second disease progression. Results from our NGS panel were compared against plasma testing with BEAMing dPCR regarding the *RAS* gene status. The overall percent of agreement was 83.6%, with a positive and negative percent agreement of 74.3% and 96.2%, respectively. Further comparison of plasma NGS with standard tissue testing used in the clinic showed an overall percent agreement of 86.7% for *RAS* status, with a positive and negative percent agreement of 81.2% and 92.8%, respectively. Thus, our study strongly supports the validity and efficiency of an affordable targeted NGS panel for the detection of clinically relevant mutations in patients with mCRC.

## 1. Introduction

Despite recent significant advances in therapeutic management, colorectal cancer (CRC) ranks among the most frequent and lethal neoplasms worldwide [1,2]. About 25% of CRC patients are already metastatic at the time of initial diagnosis, while approximately 40% of them will develop metastases after first-line treatment, accounting for the high mortality rates of the disease [3].

Targeted treatments have been shown to significantly improve survival in patients with metastatic CRC (mCRC). These are only effective in a subset of tumors with specific molecular characteristics, thus rendering tumor molecular profiling a mandate in the clinical setting [4]. In clinical practice, biomarker analysis is routinely performed on archival tissue. This includes testing for *RAS* and *BRAF* mutations prior to administration of monoclonal antibodies targeting the epidermal growth factor receptor (EGFR). To date, two anti-EGFR monoclonal antibodies, cetuximab and panitumumab, are approved for the treatment of *KRAS* and *NRAS* wild-type CRC [4]. Based on the results of large-scale phase III clinical trials, a significant clinical benefit for all-*RAS* wild type tumors has been established upon administration of these antibodies, while a detrimental effect is possible, when given to *RAS* mutated tumors [5,6,7]. Consequently, current guidelines recommend against using anti-EGFR antibodies in confirmed *RAS* mutated CRC tumors [4]. *BRAF* mutations, predominantly *BRAF* V600E, occurring in 8–12% of CRC cases, have been associated with worse prognosis and lower responses to anti-EGFR monoclonal antibodies, although this remains controversial [8,9]. *BRAF* V600E mutations also predict response to encorafenib, a recently approved potent *BRAF* inhibitor, in combination with cetuximab for patients carrying this mutation after first line treatment failure [10]. More recently, *KRAS* G12C inhibitors sotorasib and adagrasib have shown promising results in phase I/II trials and are currently being investigated in phase III trials in patients carrying this mutation [11,12].

Besides primary resistance to anti-EGFR treatment, exposure to anti-EGFR therapy can lead to the development of secondary resistance. One of the best-established mechanisms is the development of mutations in the EGFR extracellular domain (ECD), occurring in approximately 20% of CRCs following EGFR blockade, which interfere with antibody binding, leading to tumor relapse [13,14,15,16,17].

A number of other genes are also implicated in anti-EGFR resistance. *ERBB2*, which encodes a member of the EGFR receptor tyrosine kinase family, is another gene with possible therapeutic implications in mCRC [18]. Mutations in *ERBB2* correlate with increased MAPK activation and resistance to anti-EGFR antibodies [19,20]. Such mutations are known to occur in 7% of CRCs and may co-exist with *ERRB2* amplification in one fifth of cases [21]. Similar frequency was reported in a recent study that included both tissue and ctDNA cohorts, where the prevalence ranged between 4.1% and 5.8% [22]. Several phase II studies have shown the efficacy of ERBB2 blockade (trastuzumab in combination with pertuzumab, lapatinib or tucatinib) in patients with *ERBB2*-amplified mCRC [23,24,25], while *ERBB2* mutations have been associated with the response to ERBB2 blockade in patient CRC xenografts [26]. *MET* is another well-established proto-oncogene that can be altered in CRC [27]. In a series of solid tumor specimens, including 43 colon cancer samples, *MET* exon 14 skipping mutations were identified in five cases (9.3%) with colon cancer [28]. As shown in lung cancer, *MET* exon 14 skipping mutations occur mutually exclusively with other validated drivers, thus supporting its oncogenic implication and defining a distinct molecular subgroup of gastrointestinal malignancies [29]. In the preclinical analysis included in the study, both MET tyrosine kinase inhibitor and an anti-MET monoclonal antibody inhibited the growth of a patient-derived colon cancer cell line harboring *MET* exon 14 deletion [28], thus suggesting its potential role as a predictive biomarker for MET inhibitors.

The current gold standard for mutation testing to guide therapeutic options is tumor tissue material, either from biopsy or surgical resection samples. Albeit clinically useful, the information derived from tissue material provides a snapshot of the tumor that reflects a single point in space and time and does not necessarily capture intratumoral heterogeneity [29]. Furthermore, such information may become outdated, as the tumor continuously evolves and new clones emerge in response to environmental pressure, such as anti-cancer therapies. Since biopsies of recurrent or metastatic lesions are rarely performed, doctors are obliged to make therapeutic decisions without knowledge of new, potentially crucial, tumor molecular data. Evidently, real-time evaluation of actionable mutations is of utmost importance to ensure that clinical decisions are steered towards the right direction.

Liquid biopsies have emerged as promising, non-interventional techniques that have the potential to overcome the challenges of tissue profiling [30]. The circulating tumor DNA (ctDNA), which accounts for the fraction of cell-free DNA that is released from tumor cells in the blood of cancer patients, is increasingly becoming a widely used liquid biopsy biomarker [31]. ctDNA analysis can be repeated on a regular basis, providing real-time insight into the tumor’s molecular profile. As such, it can be used to monitor tumor response to therapy and to inform on the emergence of new resistant clones, empowering oncologists to make timely decisions that may improve patient outcomes [32]. 

The minute levels of ctDNA in blood circulation dictate the utilization of ultra-sensitive methods for the reliable identification of plasma mutations. These include polymerase chain reaction (PCR)-based methods such as BEAMing and droplet digital PCR, with reported detection limits as low as 0.01% [33,34]. Even though these PCR-based techniques have the required sensitivity, they present with several shortcomings that restrict their use in the clinical setting, including lack of scalability, limited coverage of interrogated hotspots and long, non-automated, costly protocols, especially in the case of BEAMing [32]. In contrast, next-generation sequencing (NGS) techniques have been gaining ground in liquid biopsy applications, as they allow for parallel detection of multiple mutations in a scalable fashion. The introduction of the use of unique molecular identifiers (UMIs) has enabled NGS techniques to reach low detection levels that, although still inferior compared to digital PCR (dPCR), may be more appropriate for clinical applications [35,36].

The aim of the present study was to develop and evaluate a sensitive, time- and cost- effective NGS assay that could be applied specifically for ctDNA analysis in metastatic CRC patients in the daily oncology practice. For this reason, our gene panel was designed based on the NCCN guidelines for CRC-associated genes and included *KRAS*, *NRAS*, *EGFR*, *BRAF*, *ERBB2* and *MET*, and covered clinically relevant hotspots. A total of 68 samples from 30 patients with metastatic CRC, collected at diagnosis and disease progression, were sequenced with this panel. Validation of the NGS assay was provided by comparing plasma data with tissue data generated during routine tumor profiling for clinical decision making. Further validation of the NGS data was provided by employing an orthogonal assay, the BEAMing dPCR, for the detection of *KRAS/NRAS* mutations.

## 2. Materials and Methods

### 2.1. Patient Enrollment and Data Collection 

Patients with a diagnosis of mCRC were enrolled in the study, which was approved by the Ethics Committee of the University Hospital of Ioannina (11/18-04-2018), after providing written informed consent. Demographic and clinical data (including tissue molecular analysis results) were prospectively collected from patient records and blood samples were collected during routine venipunctures for clinical assessment.

### 2.2. Sampling of Blood and Plasma Preparation

Samples of 2 × 10 mL of whole blood were collected in BD Vacutainer K_2_ EDTA blood collection tubes with lavender Hemogard closure or Streck cell-free DNA BCT and were processed within 4 h from collection for BD Vacutainer K_2_ EDTA tubes and within 72 h for Streck cell-free DNA BCT. For plasma isolation, blood collection tubes were centrifuged at 15 to 25 °C for 10 min at 1600× *g* using a swing bucket rotor. The isolated plasma (supernatant) was transferred to centrifuge tubes. For plasma samples from BD Vacutainer K_2_ EDTA tubes, a centrifugation at 15 to 25 °C for 10 min at 3000× *g* was performed using a fixed angle rotor, while for plasma samples from Streck cell-free DNA BCT, the isolated plasma was centrifuged at 15 to 25 °C for 10 min at 6000× *g* using a fixed angle rotor. Highly hemolytic samples were rejected. These steps were based on previously published literature and are in line with the Key Recommendations on Assay Characteristics by the United States National Cancer Institute Colon and Rectal–Anal Task Forces [32].

### 2.3. cfDNA Isolation and Quantitation

For NGS analysis cell free DNA was extracted from 4 mL plasma using the QIAamp Circulating Nucleic acid kit (QIAGEN GmbH, Hilden, Germany) and the final eluted DNA (50 uL elution volume) was quantified using the Qubit 2.0 Fluorometer (Thermo Fisher Scientific, Waltham, MA, USA) with the dsDNA high sensitivity kit (Thermo Fisher Scientific). For BEAMing Digital PCR 3 mL plasma input was used for cfDNA isolation using the same kit.

### 2.4. Library Preparation and Targeted NGS

The libraries were generated with a customized QIAseq Targeted DNA Panel Kit (QIAGEN GmbH, Germany), targeting exonic regions of KRAS (exons 2, 3 and 4), NRAS (exons 2, 3 and 4), BRAF (exon 15), ERBB2 (exons 8, 19, 20 and 21), EGFR (all exons), and MET (exon 14) based on the Homo sapiens (human) GRCh37/hg19 genome assembly, (detailed custom gene panel in Supplementary Table S3) according to the manufacturer’s protocol (as detailed in the QIAseq™ Targeted DNA Panel Handbook). Qiaseq Targeted DNA panel enables variant detection using integrated unique molecular indices (UMIs). The primers were designed to cover a region of 6787 bp. The input amount for library preparation was in the range of 8–40 ng (details in Supplementary Table S1). Briefly, for library construction, cfDNA samples were first fragmented, end-repaired and A-tailed and then the fragments were ligated with a sequencing platform-specific adapter containing UMIs and sample indexes. For final library construction a targeted enrichment PCR (using the custom designed primers and one universal primer complementary to the adapter) and a universal PCR (for library amplification and addition of platform specific adapter sequences) were conducted. Libraries were quantified by qRT-PCR using KAPA Library Quantification Kit Illumina® Platforms (Roche Diagnostics, Basel, Switzerland). Pooled libraries (10 per run) were loaded at a combined concentration of 10 pM and analyzed by paired-end sequencing on an Illumina MiSeq instrument using the MiSeq Reagent Micro kit v2 (2 × 150 bp) for up to 8 million paired-end reads using a custom sequencing primer (QIAseq A Read 1 Primer).

### 2.5. NGS Data Analysis

Data were analyzed using the QIAGEN GeneGlobe Data Analysis Center. More specifically, the read analysis workflow begins with read processing steps that (i) remove the exogenous sequences, such as PCR and sequencing adapters and UMI, (ii) identify the UMI sequence and append it to the read identifier for downstream analyses and (iii) remove short reads that lack enough endogenous sequence for mapping to the reference genome. The trimmed reads were mapped to the reference genome with BWA-MEM [37], followed by filtering of poorly mapped reads and soft-clipping of gene-specific primer sequences. The aligned reads were sent forward to variant calling through smCounter2, which is a UMI-aware low frequency variant caller available at GitHub (https://github.com/qiaseq/qiaseq-dna) (accessed between 15/09/2020 and 1/10/21) [38]. Original raw sequencing data are available at the Sequence Read Archive (SRA) with the study BioProject ID PRJNA764095.

### 2.6. BEAMing Digital PCR

To assay the samples by dPCR, the BEAMing Digital PCR was employed and the OncoBEAM™ RAS CRC CE-IVD kit (Sysmex Inostics GmbH, Hamburg, Germany) was used according to manufacturer instructions. This assay detects 34 target mutations in codons 12, 13, 59, 61, 117 and 146 of the KRAS and NRAS oncogenes against a background of wild-type genomic DNA. This assay combines dPCR with flow cytometry, achieving a detection limit as low as 0.01% [16]. More specifically, in BEAMing, each of the DNA molecules contained in a given plasma sample is departmentalized in a ‘water-in-oil’ emulsion together with a magnetic bead. Every DNA molecule hence multiplies and thousands of the identical DNA copies that arise are bound to the magnetic bead. Subsequently, DNA molecules are hybridized with tracers specific for the molecule type (derived from a wild-type or mutant allele, respectively). Qualitative and quantitative assessment is performed with flow cytometry within the initial population of DNA molecules [39].

### 2.7. Statistics

Descriptive statistics were used to correlate the sample characteristics with the NGS data and 2 × 2 contingency tables were constructed to calculate positive, negative and overall percent agreement. Quantitative correlation of NGS VAF and dPCR MAF was performed with linear regression for RAS mutations detected in ctDNA.

## 3. Results

### 3.1. Study Population

A total of 30 patients diagnosed with mCRC between February 2018 and June 2020 were included in this study. Two-thirds of the population were male, and the median age at diagnosis was 65.5 years (range 43–83). Sixteen patients (53%) had right-sided tumors, and the remaining 14 had tumors of the left colon, including the rectum. All patients received first-line systemic therapy. Twenty-one (70%) were treated with an oxaliplatin-based doublet, four with an irinotecan-based doublet and three with a triplet, whilst one patient received monotherapy and one was treated with another regimen. Chemotherapy was combined with an antiangiogenic in 15 patients (50%) and with an anti-EGFR monoclonal antibody in 11 (37%), with four patients (13%) receiving no targeted agent. A summary of the study population characteristics is provided in Table 1.

### 3.2. Development, Application and Evaluation of the NGS Assay

For the targeted NGS analysis of ctDNA samples on the MiSeq Illumina platform, we designed a custom gene panel of six CRC-associated genes (*KRAS*, *NRAS*, *BRAF*, *ERBB2*, *EGFR* and *MET*) that covered 32 known hotspots, as presented in Table 2. For library construction we applied the QIAseq Targeted DNA Panel Kit (QIAGEN), which uses the technology of the complex molecular barcodes known as UMIs that allow for the identification and removal of PCR artifacts. We used the BEAMing dPCR as a reference method to optimize our NGS assay to achieve comparable results regarding the detection of rare variants. More specifically, after preliminary experiments, we made the following adjustments to our NGS protocol that allowed us to reliably detect *KRAS/NRAS* (collectively called *RAS* from hereon) mutations with a limit of detection (LoD) ≥ 1%. The technical parameters of the NGS runs are described in Supplementary Table S1. We used the MiSeq Reagent kit v2 Micro, which allowed us to increase the average number of reads per sample from 77,000 to 535,000. We reduced the total number of samples per run to 10 and we ran together samples of similar quantity, when possible (Runs 1–7 in Supplementary Table S1). These adjustments resulted in an increase of the mean UMI coverage and the mean read depth in these runs (Supplementary Table S1), allowing us to detect all *RAS* mutations that had been detected by dPCR with a mutation allele frequency (MAF) ≥ 1%, except of two cases (Samples 22.2 and 28.1 in Supplementary Table S2). Using the dPCR as a reference method that yields true positive and true negative results, it was estimated that the NGS panel had a false positive rate of 3.8% and a false negative rate of 29% for *KRAS*. The high false negative rate is due to the fact that several samples had KRAS MAFs below the detection limit of the NGS panel (see Supplementary Table S2).

In total, we obtained and analyzed by NGS 68 samples from 30 patients at the time of diagnosis and at first disease progression (PD1), while samples were also collected and assayed at the second disease progression (PD2) for eight of these patients. For library construction, the average amount of cfDNA used per sample was 22.25 ng (range 7–40 ng) (Supplementary Table S1) with the lowest input recommended by the manufacturer being 10 ng. On average, ~6 million reads were generated per run. The average mapped reads and the mean sequencing depth per sample were 535,088 and 7871 respectively (Supplementary Table S1). The mean phred quality score (Q30, %) per run was >90% (Supplementary Table S1).

The use of samples from daily routine did not allow us to isolate an adequate amount of cfDNA in 20 samples (28.9% of cohort), resulting in a final input below 10 ng. Despite the low DNA input, our NGS assay detected *RAS* mutations in four of these samples; however, it failed to do so in five other samples that were tested positive by dPCR. Of note, only one of these five variants had an MAF higher than the LoD of the NGS assay (MAF = 2.55%, Sample 22.2 in Supplementary Table S2), with the rest having MAF ≤ 0.073% (Samples 18.1, 22.1, 25.1 and 29.1 in Supplementary Table S2). 

**Figure 1:**
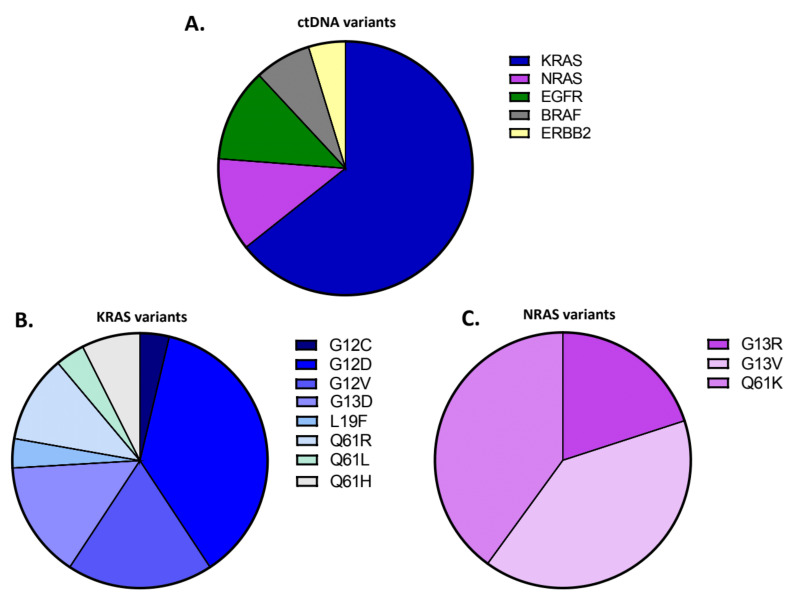
Identification of ctDNA variants in plasma of metastatic colorectal patients through the targeted NGS panel. (**A**) Distribution of the identified ctDNA variants across genes. For targeted NGS analysis in ctDNA samples, a custom gene panel of 6 CRC-associated genes (*KRAS*, *NRAS*, *BRAF*, *ERBB2*, *EGFR* and *MET*) was designed. Of the detected variants (*n* = 42), 64.3% (27/42) were detected in *KRAS*, 11.9% (5/42) in *NRAS* and *EGFR*, 7.2% (3/42) in *ERBB2* and 4.7% (2/42) in *BRAF*; (**B**) Distribution of the *KRAS* variants identified. Variant classification based on the aminoacidic change is illustrated. Of the detected *KRAS* variants (*n* = 27), G12D was the most commonly detected (37%, 10/27), followed by the G12V (18.5%, 5/27), the G13D (14.8%, 4/27), the Q61R (11.1%, 3/27), the Q61H (7.4%, 2/27) and the G12C, L19F, Q61L (3.7%, 1/27); (**C**) Distribution of the *NRAS* variants identified. Variant classification based on the aminoacidic change is illustrated. Of the detected *NRAS* variants (*n* = 5), the G13V and Q61K were identified in 40% (2/25) and the G13R in 20% (1/5).

Mutations at any time point were detected in 22 of 30 patients, with all the results of plasma testing being presented in detail in Supplementary Table S2. At baseline, a total of 15 mutations in 15 patients (50% of the patients tested) were detected. At PD1, a total of 13 mutations were detected with at least one mutation in 12 of the 30 patients (40%). In addition, one mutation was detected in seven of the eight PD2 samples. We identified mutations in 17 hotspots, with *KRAS* and *NRAS* being the most frequently mutated genes. Specifically, we identified a total of 42 variants, out of which 64.3% (27/42) were detected in *KRAS*, 11.9% (5/42) in *NRAS* and *EGFR*, 7.2% (3/42) in *ERBB2* and 4.7% (2/42) in *BRAF* (Figure 1A).

Based on these results, 50% of patients (*n* = 15) had a mutation in *KRAS*, while 10% of patients (*n* = 3) had mutations in *NRAS* (Supplementary Table S2). Among the *KRAS* variants, the G12D was the most commonly detected one (37%, 10/27) followed by the G12V (18.5%, 5/27); other detected *KRAS* variants were the G13D (14.8%, 4/27), Q61R (11.1%, 3/27), Q61H (7.4%, 2/27) and G12C, L19F, Q61L (3.7%, 1/27) (Figure 1B). Among the *NRAS* variants (*n* = 5), G13V and Q61K were identified in 40% of variants (2/5) and G13R in 20% (1/5) (Figure 1C). Moreover, the *BRAF* mutation V600E was detected in one patient in both baseline and first disease progression samples, the S784F mutation in *EGFR* was detected in another sample, and the F616L in *ERBB2* was detected in two samples (Supplementary Table S2). Notably, one EGFR variant of unspecific significance A1118T was identified, as well as three EGFR variants (C470C, I643I, Y112C) and one *ERBB2* variant V153V, which have not been previously reported (Supplementary Table S2).

### 3.3. Validation of the NGS Plasma Assay by BEAMing dPCR

In order to validate the custom targeted NGS panel, we first analyzed the concordance between the NGS assay and the BEAMing dPCR, which was used as a reference method for the detection of *RAS* ctDNA. Out of the 68 samples analyzed by NGS, 61 samples were also analyzed by BEAMin dPCR. Table 3 summarizes the concordance of *RAS* testing by the two methods. dPCR analysis detected plasma *RAS* mutations in 35/61 samples and no *RAS* mutations in 26/61 samples. NGS detected *RAS* mutations in 26/35 samples that were *RAS* mutant by dPCR (74.3% positive percent agreement) and no *RAS* mutations in 25/26 non mutant samples as per dPCR (96.2% negative percent agreement). Combining the *RAS* mutants and the *RAS* non-mutants by both methods, 51/61 concordant cases were identified (83.6% overall percent agreement or concordance) (Table 3). Cohen’s *κ* for ctDNA testing by NGS compared with dPCR was 0.68 (95% CI 0.59–0.77), thus indicative of substantial agreement according to the Landis and Koch classification [40].

**Figure 2:**
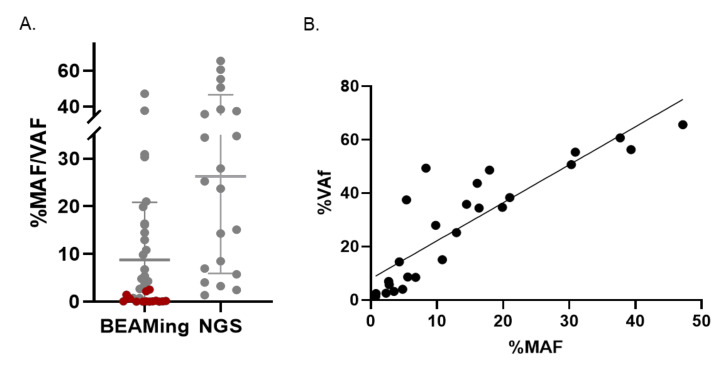
Comparison of *RAS* variants identified by BEAMing dPCR and NGS. (**A**) Scatter plot represents the mutant allele frequency (MAF) of plasma *RAS* (*KRAS/NRAS*) variants detected by BEAMing dPCR and the variant allele frequency (VAF) of these variants detected by NGS. Variants identified by both dPCR and targeted NGS are shown in grey (*n* = 21). Variants identified only by BEAMing dPCR are shown in red (*n* = 14); (**B**) Pearson correlation analysis of NGS VAF and dPCR MAF levels (*n* = 26, *R*^2^ = 0.7674, Pearson *r* = 0.8760).

Notably, there was a *KRAS* L19F mutation that was detected only by NGS (Sample 15.1 in Supplementary Table S2) and not detected by dPCR, as the latter assay did not cover this codon and was therefore not considered as a discordant case between the two methods. On the other hand, *RAS* mutant ctDNA was detected by dPCR and not by NGS in nine cases, eight of them with an MAF of 0.022–0.650%, thus, below the LoD of the NGS assay (samples in grey in Supplementary Table S2), and only one having an MAF of 2.56%. Figure 2 presents all cases with mutant ctDNA detected by at least one method (except for the *KRAS* L19F mutation that was not covered by the dPCR assay); the above-mentioned 14 mutations only detected by dPCR are shown in red in Figure 2A. The calculation of the Pearson correlation coefficient for the *RAS* variants with VAF ≥ 1% (*n* = 26) showed a good correlation between the two assays (Pearson *r* = 0.876), as shown in Figure 2B.

### 3.4. Concordance Analysis of RAS Testing in Tissue and Plasma

We evaluated the concordance of RAS testing in plasma by NGS and in tissue by standard of care tissue analysis. Only the actionable KRAS and NRAS in codons 12, 13, 59, 61, 117 and 146 were taken into account, as these are covered in standard of care tissue testing. RAS mutation status by NGS in plasma versus the standard of care tissue methods is summarized in Table 4. Plasma RAS mutations were detected in 13/16 RAS mutant tissue samples with positive percent agreement of 81.2%. No RAS mutations were detected in 13/14 non mutant tissue samples, with a negative percent agreement of 92.8%. The overall percent agreement of plasma NGS versus standard of care tissue testing for RAS was 86.7% (26/30 samples) (Table 4). Cohen’s κ for plasma ctDNA by NGS was 0.73 (95% CI 0.61–0.85) compared with tissue testing, reflecting a substantial agreement [40].

### 3.5. Association of Clinical Parameters with ctDNA

**Figure 3:**
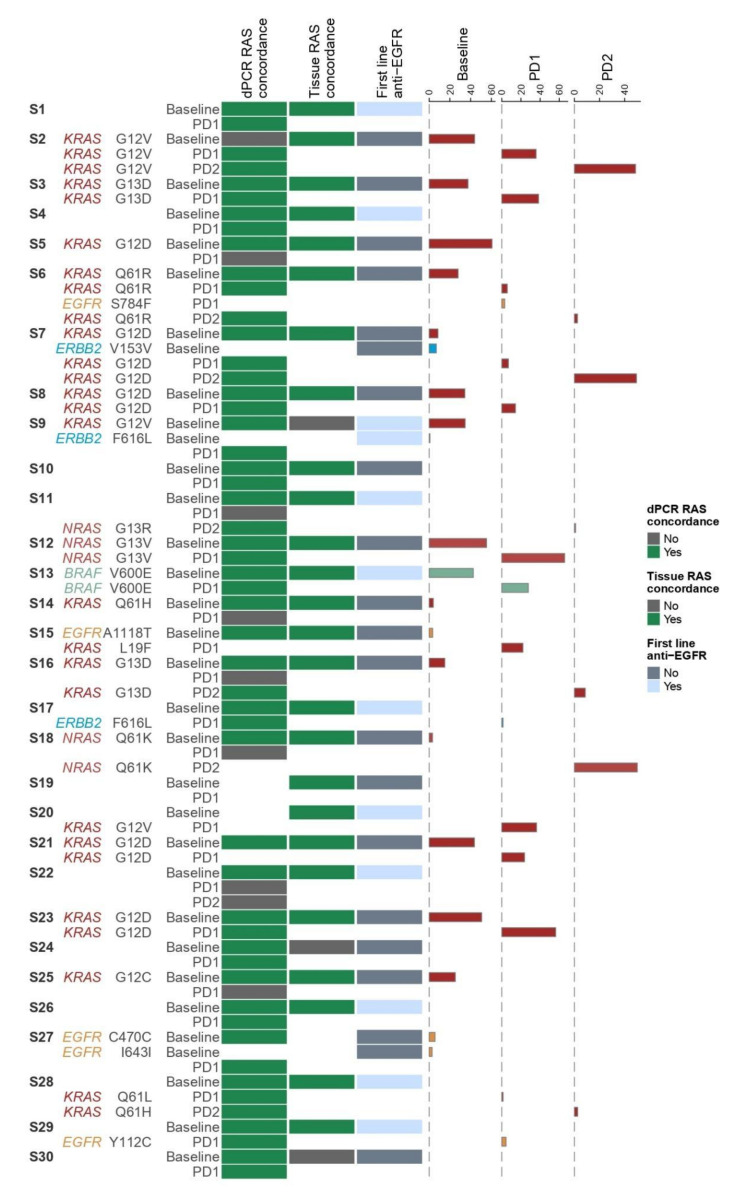
Heatmap of NGS data analysis in ctDNA in association with clinical parameters. From left to right, the patient/sample ID is mentioned (S1–S30). In case of a detected variant the gene name and the amino acid change is mentioned. Next, the disease status at sample collection (Baseline, PD1, PD2) is indicated. The first column of the heatmap, describes the concordance of *RAS* testing between NGS and dPCR (green: concordant, grey: discordant). Similarly, the second column presents the concordance of *RAS* testing in tissue and ctDNA by NGS (green: concordant, grey: discordant). In the next column, it is indicated whether the patients had received anti-EGFR therapy at baseline or not (grey: no, light blue: yes). On the right, the percentages of VAF of each variant, in each time point (Baseline, PD1, PD2) are plotted. NGS: Next-generation sequencing; dPCR: digital PCR; ctDNA: circulating tumor DNA; EGFR: Epidermal growth factor receptor; VAF: Variant allele frequency; PD1: 1st disease progression; PD2: 2nd disease progression.

Our first goal in developing and validating a multi-gene panel for mCRC was to identify additional mutations beyond RAS that can be useful to the clinician, as they are associated with therapeutic resistance to anti-EGFR in mCRC. Thus, clinical data regarding first- and second-line treatments were collected for all 30 patients of our cohort and were correlated with molecular ctDNA results, to assess whether the NGS assay could explain therapeutic responses. Overall, our panel successfully detected 11 mutant variants in genes other than RAS, two in BRAF, six in EGFR and three in ERBB2, while no mutations were detected in MET. Results for all patients are summarized in Figure 3.

Out of the five EGFR mutations that were detected by our panel, two were in the kinase domain of the EGFR, namely S784F and A1065T, in patient samples S6 and S15, respectively (Figure 3 and Supplementary Table S2). Patient S6 had a concomitant KRAS codon 61 mutation that was preserved at all sampling timepoints. This mutation had also been found in tumor tissue, thus excluding the patient from anti-EGFR therapy. Patient S15 was RAS wild type at baseline tissue, but did not receive anti-EGFR therapy. Interestingly, at first line disease progression (PD1), our targeted NGS panel detected an ‘atypical’ KRAS mutation, namely L19F. This mutation has been described to have a potential intermediate phenotype, associated with increased RAS pathway signaling, but limited oncogenic potential and continued response to cetuximab [41]. This ‘anti-EGFR naïve’ patient was challenged with anti-EGFR at second line, based on the baseline RAS wild-type status, and was progression-free at 4 months of treatment before being lost to follow-up.

The only mutation in the EGFR ECD was Y112C, which was detected in the PD1 sample of a patient with an RAS wild type tumor, who received first-line anti-EGFR therapy (S29 in Figure 3 and Supplementary Table S2). However, the Tyr112 residue is in domain I of the EGFR ECD, and not in domain III, where the well-established resistance mutations are detected [13,14,15,16,17]. The identification of Y112C upon progression after anti-EGFR therapy makes it a candidate harbinger of resistance. Our targeted NGS panel detected two additional EGFR variants, namely C470C and I643I, in the baseline sample of another patient (S27 in Figure 3). These are both synonymous, thus not causing any change in the amino acid sequence, and were detected in the baseline sample and not in the PD1 sample.

The ERBB2 F616L mutation that was detected in two patients’ samples is not considered a ‘classic’ activating mutation in non-small cell lung cancer [42]. One was detected in the baseline sample of a patient with a concomitant KRAS mutation (S9 in Figure 3 and Supplementary Table S2). This patient received anti-EGFR therapy at first line based on negative tissue RAS testing. Interestingly, both the ERBB2 and KRAS mutant clones were not detected in ctDNA at PD1. The other ERBB2 F616L mutation was detected in a patient with RAS wild type tissue after first line anti-EGFR therapy (S17 in Figure 3 and Supplementary Table S2) at PD1. However, this patient also developed a KRAS mutation only detected by dPCR, which is more likely to be responsible for the patient’s progression to anti-EGFR therapy. In light of the above, the clinical significance of the ERBB2 F616L in mCRC remains unclear. Our targeted NGS panel detected an ERBB2 synonymous variant in the baseline sample of patient with a concomitant KRAS mutation (S7 in Figure 3).

Regarding BRAF testing, a V600E mutation was detected in patient 13 at baseline (Supplementary Table S2 and Figure 3). Wild-type BRAF has long been known to be required for a response to anti-EGFR therapy [43]. However, as the BRAF status was not available from baseline tissue for this patient, anti-EGFR was administered at first line, leading to a partial response. Upon further plasma testing at PD1, the mutation was preserved, which was expected, as it is an oncogenic driver in colorectal cancer [44].

Finally, it is noteworthy that when we correlated the NGS *KRAS* VAFs with treatment lines, i.e., the times of recurrences or disease progression (PD), we observed that the median VAF at baseline was the highest and decreased with subsequent treatment lines (Supplementary Figure S1). These results are comparable with those by Elez et al. [45]. 

### 3.6. Cost and Turn-around Time of NGS Assay versus dPCR

Despite the clinical value of ctDNA testing, there is a plethora of challenges regarding the cost and implementation of liquid biopsy tests in a clinical laboratory [46]. Keeping this in mind, we developed a targeted, easy-to-use NGS assay for the monitoring of patients with mCRC. Compared to BEAMing dPCR, our NGS assay is less sensitive, as it has an LoD ≥ 1%, which is, however, an acceptable cut-off for clinical diagnostics in cancer [47]. Besides the fact that the NGS assay allows for interrogation of more hotspots than the dPCR, it also holds other advantages. The NGS assay can be easily employed, as it requires standard molecular biology techniques for library preparation, in any clinical laboratory and it can be completed in less than three days (for 10 samples). On the other hand, the BEAMing dPCR can be performed only in accredited centers by highly trained certified users and it usually requires four days to run six samples. Another important benefit of this NGS assay is that it allows for data analysis and interpretation through an automated free online tool, which yields a ready-to-use report without the need of any additional bioinformatic analysis. Finally, the NGS panel described here is cost-efficient, as the total cost per sample is ~200€ compared to the BEAMing dPCR, where the cost is ~600€ per sample. However, we should note that the OncoBEAM™ RAS CRC kit that was used for the BEAMing dPCR carries a CE-IVD mark, where the NGS assay is an in-house developed one.

## 4. Discussion

Current guidelines for the therapeutic management of patients with mCRC recommend *KRAS*, *NRAS*, *BRAF* and high microsatellite instability (MSI-H) profiling on tumor tissue samples [4]. Among those, the MSI-H phenotype is conserved throughout the natural history of CRC and can be identified with immunohistochemical approaches. A small number of other genes, such as *ERBB2* and *MET*, may also harbor actionable mutations and could expand the repertoire of interrogated genetic loci with clinical potential. The molecular status of these genes may change as a response to targeted therapies currently licensed for mCRC patients; therefore, the standard of care analysis of baseline tissue may not always reflect the actual status of these genes in subsequent treatment lines. However, obtaining an up-to-date tissue sample in mCRC patients by invasive biopsies is not feasible. ctDNA-based mutational testing presents an attractive alternative, with the potential to change the current application of precision oncology in mCRC that has yet to be approved in this setting [32]. The non-invasive nature of ctDNA sampling allows for serial testing of patients and the detection of new resistant clones in real time. Given its high diagnostic accuracy, *RAS* testing in ctDNA can be used to guide therapeutic decisions in mCRC [48]. In a landmark study, Misale et al. had reported the detection of plasma *KRAS* mutations in mCRC patients treated with anti-EGFR antibodies up to 10 months prior to the radiological disease progression [49].

We designed and employed a small NGS gene panel for the detection of ctDNA aimed to provide clinically useful information for the management of mCRC patients in a timely and cost-effective manner. Τhe panel was based on COSMIC data covering clinically relevant hotspots in five CRC-associated genes (*KRAS*, *NRAS*, *BRAF*, *ERBB2* and *MET*) and all *EGFR* exons. We selected the MiSeq benchtop sequencer (Illumina) as the running platform, which is an inexpensive system, appropriate for targeted sequencing that can be easily incorporated in the clinical setting. Another prerequisite fulfilled by this platform was that sequencing data would not require expert bioinformatic analysis and that clinically useful data could be generated shortly after the end of the run. It should also be noted that the NGS assay detected variants of unknown significance, which may prove to be clinically important in the future, as opposed to dPCR, which only analyzes a limited number of predefined mutations. 

In our study, we validated this NGS panel against plasma dPCR in a cohort of 30 patients with mCRC from Northwestern Greece with available routine clinical samples at baseline and first disease progression (PD1), while samples from eight patients were also available at second disease progression (PD2) (total of 68 samples). *RAS* status from standard of care tissue analysis at baseline was also available for all patients. Our cohort included 14 tissue *RAS* mutant patients and 16 *RAS* wild type. The NGS assay displayed acceptable concordance with both plasma dPCR (83.6%) and standard of care tissue analysis (86.7%). Additionally, compared to dPCR, the NGS assay was shown to have acceptable correlation in quantification of the detected variants expressed as MAF and VAF in dPCR and NGS, respectively (Pearson *r* = 0.8760).

Regarding the optimal cut-off for *RAS* MAF for ctDNA, this has yet to be defined [50]. Studies of tissue *RAS* testing argue that 5% is a clinically relevant cut-off for the detection of *RAS* mutations, as reducing it to 1% was not associated with better outcomes with anti-EGFR therapy [51,52]. ctDNA studies to date refer to either the cut-off of the platform used (between 0.01–1% for dPCR [53,54,55] and 1–2% for NGS [56] or to the established tissue thresholds. Interestingly, Elez et al. set 5.8% as the *RAS* MAF cut-off that best correlates with prognosis in the first and the second line of treatment [45]. However, it remains to be answered whether the same cut-off can be used to safely select the ‘clinically *RAS* wild-type’ patient population who are likely to respond to anti-EGFR therapy. It is also not clear if different *RAS* MAF cut-offs impact on the duration of response to anti-EGFR therapies. In our study, out of the 43 detected mutations, the vast majority are above the 1% cut-off (*n* = 40; 93%) and about three-quarters are above the 5% cut-off (*n* = 31; 72%). Notably, in our cohort, only a minority of the *RAS* mutations were below these cut-offs (one *NRAS* mutation in the PD2 sample has VAF < 1%, one baseline *KRAS*, one baseline *NRAS*, and one PD1/PD2 mutation in *KRAS* in the same patient have VAF < 5 %).

Results of the NGS assay were analyzed regarding their potential utility in informing therapeutic decisions in patients with mCRC. Apart from defining the *RAS* status in the plasma of newly diagnosed mCRC patients, we used our NGS panel to characterize the tumor at disease progression. Resistance to anti-EGFR monoclonal antibodies in mCRC may develop because of resistant subclones that either pre-exist or emerge due to selective pressure of the administered treatment. Although these subclones cannot be captured by tissue testing, since tissue biopsy is not indicated at this time point, they can be potentially detected in ctDNA during treatment.

In our cohort, 11 of the total 30 patients received first line anti-EGFR monoclonal antibodies cetuximab or panitumumab. In 10 of them, ctDNA testing by the NGS panel was in agreement with the *RAS* wild type status in tissue, and only one patient was found to harbor a *KRAS* and *ERBB2* mutant subclone in ctDNA that was detected by targeted NGS despite not being detected in tissue (S9 in Figure 3). This patient failed to respond to anti-EGFR therapy and progressed at three months, which is in keeping with the knowledge that *RAS* mutations are a negative predictive factor for response to the therapeutic inhibition of EGFR [4]. It also stresses the strength of ctDNA over tissue analysis for *RAS* status; tissue biopsy samples only a small area of the tumor and therefore, it cannot capture tumor heterogeneity, in contrast to ctDNA which may originate from any part of the tumor. It is of note, however, that no mutation was detected in this patient’s ctDNA at PD1. 

NGS retesting at PD1 of the 11 aforementioned patients, who received first line anti-EGFR therapy, revealed new mutations in six cases at PD1. One was in *EGFR* codon 112 (S29 in Figure 3) and two in *ERBB2* codon 616 (S9 and S17 in Figure 3), none of which is known to confer resistance to anti-EGFR therapy. The other three were in *RAS* (two in *KRAS* codon 12 and one in *KRAS* codon 61; S7, S20 and S28 in Figure 3), with one at VAF below 5%, meaning that anti-EGFR may be of benefit in this case (S28 in Figure 3). Thus, with the exception of two patients with *KRAS* mutant ctDNA over 5%, the remaining eight patients could be considered for anti-EGFR rechallenge at the second treatment line based on the ctDNA *RAS* status by NGS. 

Nineteen patients did not receive anti-EGFR treatment at first line, with fifteen being *RAS* mutated in tissue and therefore ineligible for anti-EGFR treatment, and four receiving other treatments (S10, S15, S19 and S27 in Figure 3). Of the fifteen patients with *RAS* mutations in tissue at baseline, thirteen also had *RAS* mutated ctDNA as per NGS analysis at baseline, and eight of them preserved these mutations in ctDNA at PD1. 

In contrast, a total of seven patients had no *RAS* mutation in plasma as per NGS analysis at PD1. Two of them were *RAS* mutated in tissue, but not in plasma by NGS at baseline (S24 and S30 in Figure 3). Interestingly, both patients had metastatic disease in the peritoneum, which is associated with lower levels of ctDNA shedding in the blood stream and possibly negative blood-based testing [30,32]. However, both remained *RAS* wild type in PD1. In contrast, five were tissue and ctDNA mutated by NGS at baseline, but ‘lost’ their mutation and became ctDNA wild-type by NGS at PD1 (S5, S14, S16, S18 and S25 in Figure 3), thus constituting a subgroup of possible candidates for second-line anti-EGFR therapy. This finding suggests that ctDNA testing with NGS can identify patients with *RAS* wild-type status, either at baseline or PD1, who can possibly benefit from anti-EGFR treatment. This is a promising finding that needs to be validated in the setting of clinical trials. Interestingly, out of the seven patients who could potentially benefit from anti-EGFR treatment based on NGS ctDNA testing, dPCR identified a very low amount of *RAS* mutant ctDNA, with MAF below 0.65% in five patients, which is below the NGS cut-off and are likely not to represent clinically significant clones or affect initial response to anti EGFR antibodies. Therefore, our NGS panel is able to identify the *RAS* mutant clones in clinically significant amounts.

One patient with *RAS* wild-type tissue at baseline (S15), who had not received anti-EGFR at first line, received cetuximab at second line and responded for at least four months before being lost to follow-up. Interestingly, ctDNA testing by NGS at PD1 had revealed a new *KRAS* mutation in codon 19 with VAF 22%. As this specific mutation is not associated with resistance to anti-EGFR, the fact that second-line treatment was selected based on the baseline tissue *RAS* status did not affect response in this case. Among the remaining three patients with baseline tissue *RAS* wild-type status who did not receive first-line anti-EGFR, two received second line chemotherapy only (S10 and S27), with poor responses (disease progression or death at less than 3 months), or chemotherapy with the anti-VEGF aflibercept (S19) with maintained response for over 6 months. Based on the ctDNA testing by NGS at PD1, no *RAS* mutations were detected, and all three would therefore be eligible for anti-EGFR treatment at second line, potentially achieving a longer response compared to receiving chemotherapy only.

Interestingly, our panel detected a *KRAS* G12C mutation in a baseline plasma sample (S25 in Figure 3). This mutation corresponds to a glycine-to-cysteine substitution that occurs in approximately 3% of colorectal cancers and is targeted by newly developed agents such as sotorasib and adagrasib [12]. Recent studies using these agents have shown promising results in mCRC patient cohorts, but with less prolonged responses as compared to non-small cell lung cancer, likely due to the early secondary resistance in CRC [11]. These are mainly acquired alterations of the *RAS/RAF* pathway, including activating mutations in the *KRAS*, *NRAS* and *BRAF* genes covered by our panel [57]. Of note, the *KRAS* G12C mutation detected by our NGS panel was not present in the repeat plasma sample at PD1, questioning the sequence of targeted therapies in order for patients to benefit from them, especially given that the drug approvals are based on pretreated patients at later lines of therapy.

The main limitation of our study is its sample size, as a larger sample size could possibly reveal even higher concordance of our NGS panel with tissue and dPCR. Moreover, it would allow for a higher detection rate of *BRAF* mutations and of resistance mutations at PD1/PD2 in all genes of our panel, including *MET*.

## 5. Conclusions

With liquid biopsies being steadily established as an integral part of modern oncology practice, the detection of ctDNA has multifaceted roles in the clinic and can be achieved through the application of ultrasensitive dPCR-based methodologies or specialized NGS approaches (such as TAm-Seq, Safe-SeqS and CAPP-Seq) [58]. These techniques, however, require highly trained personnel, expensive equipment and/or complex bioinformatics analysis that are often beyond the budget and human resources of a clinical laboratory. As a viable alternative, we designed and validated an NGS-based liquid biopsy test, able to detect the mutations required by the ESMO guidelines for the management of mCRC and to define the status of a small number of other genes of high clinical relevance. Our NGS assay can be run on the affordable MiSeq platform and it yields ready-to-report clinical data in less than 3 days. Despite having lower sensitivity than dPCR, our assay can effectively detect the mutant subclones represented in significant relative frequencies in ctDNA. Thus, it can readily be used to guide therapeutic decisions as soon as ctDNA-based testing is approved in mCRC outside the setting of clinical trials. Finally, the NGS assay allows for the detection of new variants and can be used to increase the amount of data for research and clinical purposes. By testing a larger number of patients in multiple time points, it can generate very important data for the evolution of mCRC, as well as for the course of the disease in individual patients in response to treatment.

## Figures and Tables

**Table 1:** Demographic and clinical characteristics of the enrolled mCRC patients.

Patient Characteristics	*n* (%)
Gender	
Male	20 (67%)
Female	10 (33%)
Age in years	
Mean	68
Range	43–83
Primary tumor location	
Right colon	16 (53%)
Left colon/Rectum	14 (47%)
First line chemotherapy	
Oxaliplatin-based doublet	21 (70%)
Irinotecan-based doublet	4 (13%)
Other ^1^	5 (17%)
First line targeted agent	
Anti-angiogenic	15 (50%)
Anti-EGFR	11 (37%)
None	4 (13%)
Total	30 (100%)

^1^ Triplet, single agent or other.

**Table 2:** List of the genes and hotspots included in the targeted NGS panel.

Gene	Exon(s)	Codon Position	Number of Hotspots
*KRAS*	2	12, 13	2
	3	59, 61	2
	4	117, 146	2
*NRAS*	2	12, 13	2
	3	59, 61	2
	4	117, 146	2
*MET*	14	1003	1
*BRAF*	15	600	1
*ERBB2*	8	309, 310	2
	19	740, 755, 767, 769	4
	20	773, 776, 777	3
	21	824, 842, 874	3
*EGFR*	All exons (1–28) including 12	451, 464, 465, 467, 491, 492	6

**Table 3:** Concordance of plasma *RAS* testing between NGS and BEAMing dPCR (*n* = 61).

	Plasma ctDNA *RAS* Status by dPCR						
	*RAS*	Mutant	WT	Total	PPA	NPA	OPA
Plasma ctDNA *RAS* status by NGS	Mutant	26	1	27	100 × 26/35 = 74.3%	100 × 25/26 = 96.2%	100 × 51/61 = 83.6%
	WT	9	25	34			
	Total	35	26	61			

ctDNA: circulating tumor DNA; dPCR: digital PCR; NGS: Next-generation sequencing; WT: Wild type; PPA: Positive percent agreement; NPA: Negative percent agreement; OPA: Overall percent agreement.

**Table 4:** Concordance of plasma ctDNA by NGS and standard tissue *RAS* testing (*n* = 30).

	Tissue *RAS* Status						
	*RAS*	Mutant	WT	Total	PPA	NPA	OPA
Plasma ctDNA *RAS* status by NGS	Mutant	13	1	14	100 × 13/16 = 81.2%	100 × 13/14 = 92.8%	100 × 26/30 = 86.7%
	WT	3	13	16			
	Total	16	14	30			

ctDNA: circulating tumor DNA; dPCR: digital PCR; NGS: Next-generation sequencing; WT: Wild type; PPA: Positive percent agreement; NPA: Negative percent agreement; OPA: Overall percent agreement.

## Data Availability

Original raw sequencing data are available at the Sequence Read Archive (SRA) with the study BioProject ID PRJNA764095.

## Supplementary Materials

The following are available online at https://www.mdpi.com/article/10.3390/diagnostics11122375/s1, Supplementary Figure S1: Correlation of NGS KRAS/NRAS VAFs with disease recurrence, Supplementary Table S1: NGS quality parameters, Supplementary Table S2: Results of plasma and tissue mutation analysis, Supplementary Table S3: Targeted NGS panel.
