# Supplementary material for: Development and Validation of a Targeted ‘Liquid’ NGS Panel for Treatment Customization in Patients with Metastatic Colorectal Cancer
Source: Diagnostics (Basel). 2021 Dec 16;11(12):2375. doi: 10.3390/diagnostics11122375 (PMC8700616; doi:10.3390/diagnostics11122375)

Supplementary Materials

Development and validation of a targeted 'liquid' NGS panel for treatment customization in patients with metastatic colorectal cancer

Myrto Kastrisiou, George Zarkavelis, Anastasia Kougioumtzi, Prodromos Sakaloglou, Charilaos Kostoulas, Ioannis Georgiou, Anna Batistatou, George Pentheroudakis and Angeliki Magklara

Supplementary Figure S1: Correlation of NGS VAFs for KRAS/NRAS with disease recurrence. NGS VAFs for KRAS/NRAS were correlated with treatment lines, i.e., the times of recurrences or disease progression (PD). 1st line corresponds to baseline, 2nd line to PD1 and 3rd line to PD2. In samples with more than one variants the highest VAF was selected.

Supplementary Table S1. NGS quality parameters. For NGS analysis of cfDNA samples, Pooled libraries (10 per run) were loaded at a combined concentration of 10 pM and analyzed by paired-end sequencing on a Illumina MiSeq instrument using the MiSeq Reagent Micro kit v2 (2×150 bp) for up to 8 million paired-end reads using a custom sequencing primer (QIAseq A Read 1 Primer). NGS quality parameters are presented in the table for each Run (Run 1-7).

Supplementary Table S2. Results of plasma and tissue mutation analysis. In total, we obtained and analyzed by NGS 68 samples from 30 patients at the time of diagnosis and at first disease progression (PD1), while samples were assayed at the second disease progression (PD2) for eight of these patients. Results of cfDNA analysis by NGS and BEAMing digital PCR (only RAS testing) are presented, and also results from tissue RAS testing at baseline in all patients. In samples in grey and bold, RAS variants have detected only by BEAMing PCR.

Supplementary Table S3. Targeted NGS panel. NGS targeted panel was designed to cover exonic regions of KRAS (exons 2, 3 and 4), NRAS (exons 2, 3 and 4), BRAF (exon 15), ERBB2 (exons 8, 19, 20 and 21), EGFR (all exons), and MET (exon 14). Panel design was conducted using QIAGEN GeneGlobe Custom software based on the Homo sapiens (human) GRCh37/hg19 genome assembly.

Supplementary Figure S1

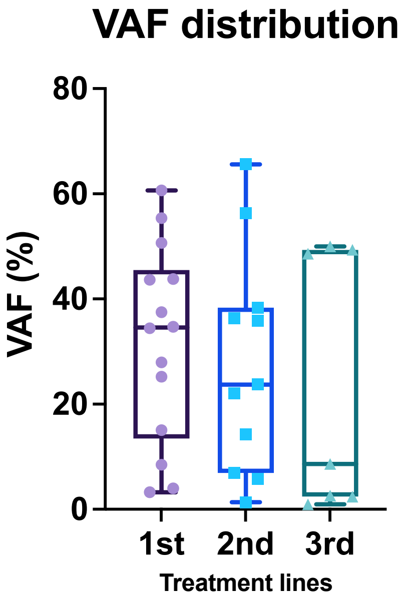

Supplement: Supplementary file 1 [file diagnostics-11-02375-s001.zip › Supplementary Materials.pdf]
